# Long-Term Results of Endoscopic Metal Stenting for Biliary Anastomotic Stricture after Liver Transplantation

**DOI:** 10.3390/jcm12041453

**Published:** 2023-02-11

**Authors:** Aymeric Becq, Alexis Laurent, Quentin De Roux, Cristiano Cremone, Hugo Rotkopf, Yann Le Baleur, Farida Mesli, Christophe Duvoux, Aurélien Amiot, Charlotte Gagniere, Nicolas Mongardon, Julien Calderaro, Daniele Sommacale, Alain Luciani, Iradj Sobhani

**Affiliations:** 1 Department of Gastroenterology, Henri Mondor Hospital—APHP and Paris Est Creteil University, 1 Rue Gustave Eiffel, 94000 Creteil, France; 2 Department of Digestive Surgery, Henri Mondor Hospital—APHP and Paris Est Creteil University, 1 Rue Gustave Eiffel, 94000 Creteil, France; 3 Intensive Care Unit, Henri Mondor Hospital—APHP and Paris Est Creteil University, 1 Rue Gustave Eiffel, 94000 Creteil, France; 4 Department of Hepatology, Henri Mondor Hospital—APHP and Paris Est Creteil University, 1 Rue Gustave Eiffel, 94000 Creteil, France; 5 Department of Pathology, Henri Mondor Hospital—APHP and Paris Est Creteil University, 1 Rue Gustave Eiffel, 94000 Creteil, France; 6 Department of Radiology, Henri Mondor Hospital—APHP and Paris Est Creteil University, 1 Rue Gustave Eiffel, 94000 Creteil, France

**Keywords:** endoscopic retrograde cholangiopancreatography (ERCP), biliary anastomotic stricture, liver transplantation

## Abstract

(1) Background: Anastomotic biliary stricture (ABS) is a well-known complication of liver transplantation which can lead to secondary biliary cirrhosis and graft dysfunction. The goal of this study was to evaluate the long-term outcomes of endoscopic metal stenting of ABS in the setting of deceased donor liver transplantation (DDLT). (2) Methods: Consecutive DDLT patients with endoscopic metal stenting for ABS between 2010 and 2015 were screened. Data on diagnosis, treatment and follow-up (until June 2022) were collected. The primary outcome was endoscopic treatment failure defined as the need for surgical refection. (3) Results: Among the 465 patients who underwent LT, 41 developed ABS. It was diagnosed after a mean period of 7.4 months (+/−10.6) following LT. Endoscopic treatment was technically successful in 95.1% of cases. The mean duration of endoscopic treatment was 12.8 months (+/−9.1) and 53.7% of patients completed a 1-year treatment. After a mean follow-up of 6.9 years (+/−2.3), endoscopic treatment failed in nine patients (22%) who required surgical refection. Conclusions: Endoscopic management with metal stenting of ABS after DDLT was technically successful in most cases, and half of the patients had at least one year of indwelling stent. Endoscopic treatment long-term failure rate occurred in one fifth of the patients.

## 1. Introduction

Anastomotic biliary stricture (ABS) is the most common cause of biliary obstruction in the setting of liver transplantation (LT) [1,2]. ABS accounts for roughly 40% of all biliary complications. Although the occurrence of ABS has decreased over the last decade, it remains an issue with a reported incidence of 4–9% [3]. The cumulative estimated risk of biliary stricture is approximately 6.6%, 10.6%, and 12.3%, within 1, 5 and 10 years post-LT, respectively [4]. The majority of ABS occur within the first year after LT. However, ABS can be seen later on, as well [4]. Magnetic Resonance Cholangiography (MRC) is the best diagnostic procedure when ABS is suspected, usually in the setting of abnormal liver function tests (LFTs), with a sensitivity and specificity of 90% [5]. In case of findings suggestive of ABS and persistently elevated LFTs (>4 weeks), treatment is recommended by the European Society of Gastrointestinal Endoscopy (ESGE) guidelines [6].

Untreated ABS can lead to secondary biliary cirrhosis and graft dysfunction. Endoscopic retrograde cholangiopancreatography (ERCP) is the preferred method of treatment [7]. The 2018 ESGE guidelines suggest temporary insertion of multiple plastic stents. The admitted total duration of treatment is usually 12 months [6]. The European Association for the Study of the Liver (EASL) guidelines also suggest ERCP with balloon dilatation and stent placement as a first-line therapy [8]. When endoscopic treatment fails, hepaticojejunostomy should be discussed.

The modalities of stenting such as the optimal type of stent, timing and duration are still debated to date. Fully covered self-expending metal stents (FC-SEMS) are now used in many centers as the number of ERCPs needed to achieve stricture resolution is lower [6,9]. Compared to multiple plastic stents, data are conflicting with some studies suggesting a higher resolution rate and a lower recurrence rate with the latter [10,11]. Furthermore, risk factors of failed endoscopic treatment are not well known. Finally, long-term results after endoscopic management have been studied, specifically in the setting of living donor liver transplantation (LDLT), often performed in Asian countries. In France, deceased donor liver transplantation (DDLT) is practiced in most cases; although the recurrence risk seems lower [12], long-term data, specifically regarding metal stenting, is scarce.

The goal of our study was to evaluate the long-term outcomes of the endoscopic management of ABS by metal stenting in the setting of DDLT in a tertiary center.

## 2. Materials and Methods

All consecutive patients with ABS secondary to LT were identified. Patients with LDLT and ABS treated exclusively by plastic stenting were excluded. ABS was defined as a stricture identified on MRCP in patients with persistently elevated LFTs or jaundice. A stricture was defined as a significant discrepancy in duct caliber between the native and the donor duct, with proximal intrahepatic duct dilatation (i.e., visible beyond the second biliary confluence). Bile duct dilatation was defined as a measured diameter of ≥ 6 mm, adjusted for patients older than 60 years (+1 mm for every 10 years of age) [13]. Persistently elevated LFTs was defined as a value of > 1.5 of the upper normal limit for over 4 weeks. In our institution, patients with LT underwent routine clinical evaluation and LFTs every month during the first 6 months, and then every 3 months. All cases of diagnosed ABS were discussed in a multi-disciplinary staff meeting prior to treatment. Endoscopic management was the preferred choice of treatment. ERCP and surgical procedures were performed according to standard clinical practice. The follow-up period was up to June 2022. Given the observational and retrospective nature of the study, patient consent was waived as per French law. In addition, the study was conducted in accordance with the MR004 procedure from the national commission of data processing and freedoms regarding non-interventional studies (CNIL, N° 1,348,478 and 1,311,293). The study protocol conforms to the ethical guidelines of the 1975 Declaration of Helsinki (6th revision, 2008).

### 2.1. Liver Transplantation

LT technique has been reported previously [14,15]. Briefly, LT was performed using preservation of the inferior vein cava (IVC) and temporary portacaval anastomosis except when IVC resection was necessary. Reconstruction was performed in the piggyback fashion with end-to-side cava-caval anastomosis on the joined stump of the three main hepatic veins. Portal, arterial and biliary reconstructions were performed using standard techniques.

### 2.2. Endoscopic Management

All ERCPs were performed with general anesthesia by expert therapeutic endoscopists. Antibiotic prophylaxis and preventive measures for post-ERCP pancreatitis was routinely administered. Deep bile duct cannulation was performed with a sphincterotome (Olympus^®^ KD-V211M-0725, Tokyo, Japan), pre-loaded with a hydrophilic-tipped guidewire (0.025 inch). After successful cannulation, a cholangiography was performed by contrast injection (thus confirming the ABS), followed by biliary sphincterotomy. If the distance between the stricture and the hilum (i.e., the donor duct) was greater than 1 cm, a 60 or 80 mm-long FC-SEMS (Boston Scientific Co^®^, Boston, MA, USA or Cook Medical^®,^ Bloomington, IN, USA) was placed across the stricture under fluoroscopic guidance. In case of a very early ABS (i.e., <3 weeks post-LT) or an associated bile leak, a plastic stent was placed at first before a change for a metal stent. In case of tight strictures, hydrostatic dilatation using an 8 or 10 mm large balloon catheter (Hurricane; Boston Scientific Co^®^) was performed prior to stenting. Urgent ERCP was performed in case of clinical, biological and/or radiological signs suggesting stent migration or obstruction. At the 6-month follow-up, repeat ERCP was performed for stent extraction and reevaluation. A new stent was placed for extra 6 months so as to pursue the calibration process. After one year of treatment, the stent was extracted. Calibration of the biliary anastomosis was then assessed by pulling through a biliary extraction-balloon catheter inflated up to 8.5–9 mm. Satisfactory calibration was defined as the absence of resistance during the balloon catheter pull-through at the level of the anastomosis associated with bile and contrast drainage seen endoscopically and fluoroscopically after cholangiogram. If satisfactory calibration was obtained, endoscopic treatment was terminated and no stent replacement was performed. In case of suboptimal calibration, a stent was placed for extra 6 months (18 months total). If calibration was not obtained at 18 months or in cases of cholangitis despite stenting, surgery was discussed. Recurrence of ABS was defined as a prolonged rise in LFTs (>1.5 normal upper limit, >4 weeks) associated with MRCP findings suggestive of ABS after biliary stent removal.

### 2.3. Surgical Management

In case of surgical management, Roux-en-Y (R-Y) hepaticojejunostomy or duct-to-duct biliary reconstruction were performed. The duct-to-duct biliary reconstruction was favored in case of a long biliary duct given the “S”-shaped biliary duct in these cases, causing a “syphon effect”.

### 2.4. Data Collection

We reviewed all medical files to collect the following baseline characteristics: age, gender, LT indication, Model for End stage Liver Disease (MELD)-score prior to LT, number of prior LTs, age, gender and CMV status of donor, type of graft, pre-LT cold ischemia duration, total operating time, combined transplantation, and post-LT CMV infection. We collected the following variables pertinent to ABS diagnosis: time to ABS occurrence (surgery to MRCP), associated bile leak, LFTs prior to ERCP, MCRP findings (ABS length and diameter, hilum to stricture distance, intrahepatic duct (IHD) dilatation, common left and right IHD duct size, presence of stones). We collected the following variables pertinent to endoscopic management by ERCP: ABS length and diameter, hilum to stricture distance, IHD dilatation, balloon dilatation, stent type and length, technical success, number of stent exchanges, total treatment duration, urgent ERCP for stent migration and/or obstruction, and adverse events (post-sphincterotomy bleeding, acute pancreatitis and cholangitis). After endoscopic treatment, the following variables were collected: duration of follow-up, prolonged rise in LFTs (>1.5 normal upper limit, >4 weeks), recurrence of ABS on MRCP, time to recurrence (last ERCP to diagnosis), surgical management, time to surgery (last ERCP to surgery), type of surgery, and survival.

### 2.5. Outcomes

The primary outcome of the study was endoscopic treatment failure (ETF). ETF was defined as the need for surgical management. The secondary outcomes were the recurrence of ABS, risk factors of ABS recurrence and adverse events of endoscopic treatment.

### 2.6. Statistics

Continuous variables are expressed as means ± standard deviations (SD) and were compared using the Student’s t test. Categorical variables are expressed as absolute numbers and percentages and were compared using chi-squared tests or Fisher exact tests, depending on the sample size. Univariate and multivariate conditional logistic regression analysis procedures were used to obtain crude and adjusted Odds Ratios (ORs) with 95% confidence intervals (95% CI) after controlling simultaneously for potential confounders. *p*-values less than 0.05 were regarded as statistically significant and factors associated with mortality with *p*-value setting at 0.1 or less were included in multivariate analysis using the Cox proportional hazards model.

To identify variables with a strong effect on the probability of ERCP treatment failure, we used a logistic regression model. The binary outcome of the logistic regression was the variable “endoscopic treatment failure” as previously defined.

Data managing, statistical evaluation, and analysis were performed with R software (version 4.1.0, R Foundation for Statistical Computing, Vienna, Austria). All the packages, libraries, and functions refer to the R Software

## 3. Results

### 3.1. Patients and Management

Between 2010 and 2015, 465 patients underwent LT. The mean age was 53.3 years (+/−10); 74.6% of patients were male, 88.2% of patients had history of a single LT, 99.6% had a total liver graft. The mean cold ischemia duration was 7.6 h (+/−2.2) and the mean duration of intervention was 7.6 h (+/−2.3).

**Figure 1:**
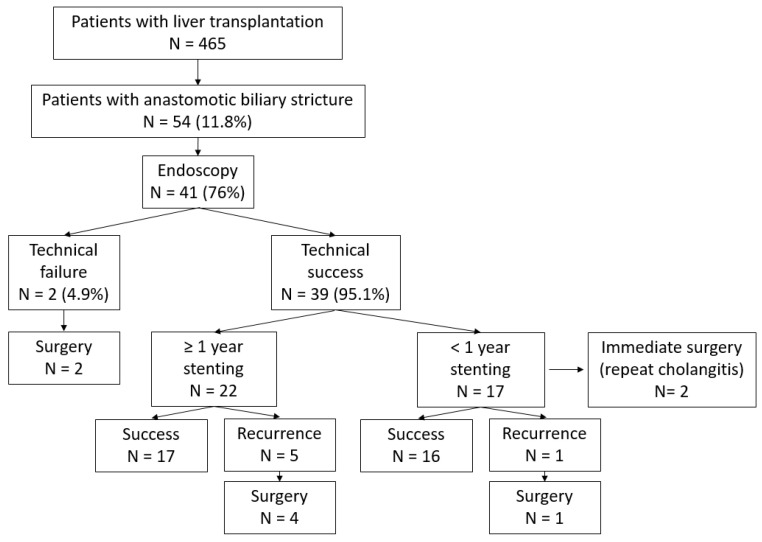
Flow chart.

Of the 465 patients, 54 (11.6%) developed an ABS requiring treatment. Amongst these patients, 41 (75.9%) were managed endoscopically (Figure 1) and included in the study. No patient was excluded due to plastic stenting alone.

Amongst the study population, the mean age at LT was 53.1 years (+/−10) and 85.4% were male. Overall, 86.8% of patients had cirrhosis, and LT main indications were HCC, HBV and HCV. The mean MELD score was 20.3 (+/−11.1). A total of 75.6% of patients were post-first LT, and 87.5% had a total liver graft. The mean donor age was 54.9 (+/−19.7). All patients had a DDLT. A CMV mismatch occurred in 36.6% of cases. The mean cold ischemia duration was 7.6 h (+/−2) and the mean duration of intervention was 7.9 h (+/−1.9). Patient’s baseline characteristics can be seen in Table 1.

ABS was diagnosed after a mean period of 7.4 months (+/−10.6) following LT. Half of the ABS cases occurred within 3 months of LT. A bile leak was associated with 10% of cases. There were no cases with non-anastomotic additional stricture. The mean total bilirubin level was 47.4 µM (+/−82.5). The mean ABS length was 10.5 mm (+/−6.6) and the mean size of the proximal CBD was 10.4 mm (+/−2.6). Baseline LFTs and MRCP findings can be found in Table 2.

ERCP was technically successful in 39 of 41 cases (95.1%). In two cases, the guidewire could not be advanced across the stricture. One patient rapidly underwent surgery with duct-to-duct biliary reconstruction. One patient was managed conservatively until prolonged abnormal LFTs prompted R-Y hepaticojejunostomy a year and a half later.

The mean ABS length was 6.5 mm (+/−4.5), and IHD dilatation was detected in 78% of cases. A FC-SEMS was used in 89.7% of patients. Am 8 cm-long stent was used in 85.7% of these cases (30/35), and a 6 cm-long stent was used in the remaining 14.3% of cases (5/35). A plastic stent followed by FC-SEMS was used in 10.3% of cases. Over the study period, the mean number of urgent ERCPs was 0.7 (+/−1.1) and the mean number of ERCPs for stent migration was 0.7 (+/−0.9). The mean total number of ERCPs was 3.8 (+/−2). The mean duration of endoscopic treatment was 12.8 months (+/−9.1). Complete calibration of the ABS was seen on last ERCP in 33 patients (80.5%). A total of 22 (53.7%) patients completed a 1-year period of biliary stenting. Stent migration with decision not to place a new stent accounts for the 17 patients with stenting for less than a year. Post-sphincterotomy bleeding occurred in four patients (9.8%), post-ERCP pancreatitis occurred in six patients (14.6%) and cholangitis occurred in two patients (4.9%). Endoscopic management details can be found in Table 3. No patient died due to ERCP-related adverse events. During follow-up, one patient died due to history of liver disease and two patients died due to unrelated causes.

### 3.2. Endoscopic Treatment Failure

After a mean follow-up of 6.9 years (+/−2.3), endoscopic treatment failed in 9/39 (22.0%) of patients. All surgical refection of the anastomosis were conducted in a mean time of 3.1 months (+/−1.3).

Five patients underwent surgery after endoscopic treatment had ended, four of which had a R-Y hepaticojejunostomy and one had a second LT because of multiple episodes of cholangitis, chronic rejection and variceal bleeding (Figure 1).

Two patients underwent surgery before endoscopic calibration was over with R-Y hepaticojejunostomy because of multiple episodes of cholangitis despite biliary stenting.

### 3.3. Recurrence of ABS and Risk Factors

ABS recurred in 6 of 37 cases (16.2%) with stent extraction (two ERCP failures and two with surgery before complete calibration). The median time to recurrence was 6 months (min 3; max 87). Four recurrences occurred within 6 months of stent removal. Five patients underwent surgery and one patient was managed conservatively, with simple surveillance (Figure 1). Logistic regression did not reveal risk factors of recurrence of ABS (Table 4). Of note, longer cold ischemia duration before LT was associated with a lower risk of recurrence of ABS, although this did not reach statistical significance (OR 2.05; *p* 0.079).

## 4. Discussion

In this monocentric cohort study, we report on the long-term results of endoscopic biliary metal stenting of ABS in the setting of deceased donor liver transplantation. We determined that endoscopic management is feasible with a 78.0% rate long-term success. Overall, 22.0% of patients ultimately required a surgical management. Furthermore, no predictive factors of endoscopic treatment failure were identified.

To our knowledge, this is the first study assessing the long-term efficacy and safety of biliary metal stenting in DDLT. First, long-term results of endoscopic stenting after LDLT have been published in multiple studies over the past 15 years [16,17]. Recently, in a 2019 study including 96 LDLT patients treated by balloon dilation as first line therapy and plastic stents for refractory cases, biliary stricture recurred in 57.9% and 4% of cases respectively, after a median follow-up of 90.9 months [18]. In a 2022 study including 639 LDLT patients with a 21.3% rate of biliary stricture occurrence and a median follow-up of 106 months, the stricture resolution rate was approximately 90% after plastic stenting (without sphincterotomy). In a study published in 2016 including 56 DDLT patients treated with multiple plastic stents and a mean follow-up of 5.8 years, the recurrence rate was 6% [19]. ABSs are more frequent following LDLT compared to DDLT and are challenging to treat, yielding a higher recurrence rate (23% vs 25% higher) [12]. Our results differ from prior data. Long-term efficacy in DDLT patients might not be better than that of LDLT. Our study provides data on long-term results of metal stenting in DDLT patients, suggesting satisfactory results which need prospective confirmation. Second, multiples studies, including three randomized controlled trials, have compared metal and plastic stents in ABS. Two reported no difference in terms of efficacy, whereas the largest study reported a higher stricture recurrence with metal stents [20]. In a review published in 2016 (601 patients), the stricture resolution rates were higher with plastic stents [10]. In our study, the mean numbers of urgent ERCPs and stent migration were consistent with (sometimes lower than) the published data [21]. While this study did not aim at comparing plastic and metal stents, it is worth noting that the overall result in terms of failed endoscopic treatment is similar between our study and published data. In all, it is uncertain whether a specific type of stent should be recommended but rather that it might depend on the length between the stricture and the hilum, the presence of an associated leak, and the recentness of LT. Overall, metal stents should probably be used whenever possible. However, given the high migration rate and subsequent need for repeat ERCPs as well as the high cost of metal stents, the cost effectiveness of the latter is unclear and warrants an evaluation. Preventive methods such as metal stents with an anti-migration system should be investigated further [22]. Third, the rates of adverse events are higher in our study compared to published data, although these results are unsurprising. A thin biliary duct is a known risk of post-ERCP pancreatitis. It is always the case in patients with an upstream biliary stricture. Cholangitis is also expected to be higher in patients undergoing immunosuppressive therapy. The rate of bleeding is, however, higher than expected, which is harder to explain here, given most patients had metal stents which is often used to stop immediate post sphincterotomy bleedings. No patient died from ERCP-related adverse events. Overall, these results suggest that although endoscopic therapy is a safe first line treatment, caution is necessary in these high-risk patients. ERCP for ABS should be performed in tertiary centers by experienced endoscopists.

Endoscopic treatment failed in 22% of cases. This is consistent with reported rates of failure of up to 30% [3,12]. Of the patients who underwent surgery, all but one had a successful outcome. In cases of failed first-line endoscopic therapy, surgery should be discussed, and remains a viable option. It is interesting to note that of the nine patients with failed endoscopic treatment, two underwent surgery as the stricture could not be passed during ERCP (patients never had a stent placed) and two underwent surgery because of recurrent cholangitis. This signifies that nearly half of the failure cases were not directly due to recurrent ABS after stent removal. It is also noteworthy that despite the fact that only half of the patients had a full-year calibration of their ABS, treatment was efficient in more than 80% of cases. Furthermore, of the five patients with failed treatment excluding technical failure and recurrent cholangitis cases, four had a full-year calibration of their ABS. Optimal duration of endoscopic treatment is still up for debate, and it is likely that it varies, some patients probably requiring a longer treatment because of recurrence risk factors. A comparative large-scale study is, however, warranted to identify the optimal cutoff suitable for most patients.

In our study, no risk factor of ABS recurrence was identified. This might be due to a lack of power as a result of a limited number of patients. Longer cold ischemia duration before LT might be associated with a lower risk of recurrence. In addition, it has been suggested by a small study including patients with chronic pancreatitis that short biliary strictures may respond better to stenting compared to longer strictures [23]. It is possible that ABS responds similarly depending on the type of anastomosis and shape of the stricture. Risk factors in the setting of LDLT patients have been identified, including older age, longer operation duration, pouched morphology of the ABS, multiple ductal anastomosis, and persistent bile leak [24,25]. Our results suggest that it might not be the case in DDLT patients, although this cannot be confirmed here. We believe that large-scale studies are warranted, as identifying risk factors would allow a tailored management. Surgical first-line management could be discussed for patients at high risk of ABS recurrence. In the meantime, given the lack of risk factors and the high long-term success rate, metal stenting seems the appropriate course of action in these cases.

Our study has several limitations inherent to the design. Although our center is a LT referral center with a large number of procedures, the number of patients enrolled was limited, probably accounting for the lack of identified recurrence risk factors. Moreover, given the lack of strong guidelines, we cannot rule out a bias in medical decision.

In conclusion, we report on the long-term results of endoscopic management with metal stenting of anastomotic strictures after deceased donor liver transplantation. Treatment was technically successful in most cases, and half of the patients had at least one year of indwelling stent. Overall, endoscopic treatment failed in one fifth of patients, most of which had subsequent surgery. No risk factor of recurrent ABS was identified. Endoscopic management in this setting is safe but should be realized in specialized, high-volume centers.

## Figures and Tables

**Table 1:** Patient baseline characteristics.

	Cohort	Failed Endoscopic Treatment	Successful Endoscopic Treatment	*p*-Value
	(N = 41)	(N = 9)	(N = 32)	
Age at LT, years	53.1 (10) (20.4; 69.9)	50.5 (10.3) (31.4; 61.5)	53.8 (9.9) (20.4; 69.9)	0.415
Gender				
Female	6 (14.6%)	2 (22.2%)	4 (12.5%)	0.597
Male	35 (85.4%)	7 (77.8%)	28 (87.5%)	
MELD score prior to LT	20.3 (11.1) (6; 40)	23.7 (7.3) (12.7; 33.2)	19.4 (11.9) (6; 40)	0.197
Number of prior LTs	1.1 (0.3) (1; 2)	1 (0) (1; 1)	1.1 (0.3) (1; 2)	0.044
LT indication				
Other	30 (73.2%)	5 (55.6%)	25 (78.1%)	0.217
Cirrhosis	11 (26.8%)	4 (44.4%)	7 (21.9%)	
Type of donor				
DDLT	41 (100%)	9 (100%)	32 (100%)	---
LDLT	0 (0%)	0 (0%)	0 (0%)	
Domino	0 (0%)	0 (0%)	0 (0%)	
Type of graft				
Full Graft	36 (87.8%)	9 (100%)	27 (84.4%)	
Right liver	5 (12.2%)	0 (0%)	5 (15.6%)	0.568
Left liver	0 (0%)	0 (0%)	0 (0%)	---
Donor age, years	54.9 (19.7) (19; 89)	64 (12.2) (50; 80)	52.2 (20.8) (19; 89)	0.043
CMV mismatch (R−/D+)				
No	26 (63.4%)	5 (55.6%)	21 (65.6%)	0.701
Yes	15 (36.6%)	4 (44.4%)	11 (34.4%)	
Pre-LT cold ischemia duration	7.6 (2) (4.1; 12.3)	7.9 (2.1) (5.6; 11.9)	7.5 (1.9) (4.1; 12.3)	0.625
Duration of LT	7.9 (1.9) (4.5; 15)	8.5 (3) (4.5; 15)	7.7 (1.4) (4.7; 10.7)	0.444

LT: liver transplantation; MELD: model for end stage liver disease; DBD-LT: donation after brain death liver transplantation; DCD-LT: donation after circulatory death liver transplantation. Data are expressed as mean +/− standard deviation (min; max), or numbers (percentage).

**Table 2:** Patients baseline LFTs and MRI findings.

	Cohort	Failed Endoscopic Treatment	Successful Endoscopic Treatment	*p*-Value
	(N = 41)	(N = 9)	(N = 32)	
LT to diagnosis delay (months)	7.4 (10.6) (0; 54)	7.2 (6.9) (0; 18)	7.5 (11.6) (0; 54)	0.937
Associated bile leak	10 (24.4%)	2 (22.2%)	8 (25%)	0.99
Baseline LFTs				
AP	332.3 (206.7) (62; 1077)	377.3 (211.2) (76; 713)	318.7 (207) (62; 1077)	0.476
GGT	598.9 (463) (44; 1639)	551.8 (448.9) (101; 1267)	613.1 (473.7) (44; 1639)	0.728
ALT	133.6 (134.9) (10; 523)	126.1 (158.3) (20; 523)	135.9 (130.1) (10; 505)	0.869
AST	85.1 (92.2) (14; 526)	65.3 (59.2) (18; 214)	91 (100.1) (14; 526)	0.35
Total bilirubin	47.4 (82.5) (3; 362)	49.4 (117.3) (5; 362)	46.8 (71.6) (3; 304)	0.95
Conjugated bilirubin	37.1 (67.8) (1; 322)	42.1 (105.1) (1; 322)	35.6 (54.4) (1; 220)	0.863
Baseline MRI findings				
Stenosis length (mm)	10.5 (6.6) (2; 27)	11 (5.7) (3; 18)	10.4 (6.9) (2; 27)	0.82
Stenosis diameter (mm)	0.6 (1) (0; 5)	0.7 (0.8) (0; 2)	0.6 (1.1) (0; 5)	0.754
Stenosis to hilum distance (mm)	23.2 (6.9) (11; 37)	25.3 (7.1) (18; 35)	22.7 (6.9) (11; 37)	0.405
Associated stone	4 (9.8)	0 (0)	4 (100)	0.043

LT: liver transplantation; LFTs: liver function tests; AP: alkaline phosphatase; GGT: gamma-glutamyl transferase; ALT: alanine transaminase, AST: aspartate transaminase; MRI: Magnetic resonance imaging. Data are expressed as mean +/− standard deviation (min; max), or numbers (percentage).

**Table 3:** Endoscopic management.

	Cohort	Failed Endoscopic Treatment	Successful Endoscopic Treatment	*p*-Value
	(N = 41)	(N = 9)	(N = 32)	
First ERCP				
Stenosis length	6.5 (4.5) (2; 20)	9.2 (7.2) (2; 20)	5.9 (3.6) (2; 15)	0.327
Stenosis diameter	3.3 (1.4) (1; 7)	2.8 (1) (2; 4)	3.4 (1.5) (1; 7)	0.234
Stenosis-to-hilum distance	24.6 (9.4) (10; 45)	30 (12.2) (15; 45)	23.8 (9) (10; 45)	0.391
IH bile ducts dilatation	32 (78)	5 (55.6)	27 (84.4)	0.07
Proximal CBD dilatation	33 (80.5)	5 (55.6)	28 (87.5)	0.07
Technical success (%)	39 (95.1)	---	---	---
Type and size of stent				
FC-SEMS	35 (84.6)	7 (100)	28 (87.5)	0.783
6 cm	5	1	4	
8 cm	30	6	24	
Plastic followed by FC-SEMS	4 (10.3)	0 (0)	4 (12.5)	
Follow-up during endoscopic treatment				
ERCPs due to stent migration				
0	19 (46.3%)	5 (55.6%)	14 (43.8%)	0.442
1	15 (36.6%)	2 (22.2%)	13 (40.6%)	
2	5 (12.2%)	1 (11.1%)	4 (12.5%)	
4	1 (2.4%)	0 (0%)	1 (3.1%)	
Number of stent exchange	1.9 (1.8) (0; 7)	1.6 (1.5) (0; 4)	1.9 (1.9) (0; 7)	0.537
Total number of ERCPs	3.8 (2) (1; 9)	3.7 (1.8) (1; 6)	3.8 (2) (2; 9)	0.804
Total treatment duration	12.8 (9.1) (0; 36)	8.7 (7.3) (0; 22)	14 (9.3) (3; 36)	0.633
Anastomotic calibration duration				
<1 year	19 (46.3%)	5 (55.6%)	14 (43.8%)	0.709
>1 year	22 (53.7%)	4 (44.4%)	18 (56.2%)	
ERCP adverse events				
Post-sphincterotomy bleeding	4 (9.8)	1	3	0.99
Acute pancreatitis	6 (15)	2	4	0.61
Cholangitis	2 (4.9)	1	1	0.41
Death	0 (0.0)	0	0	1

ERCP: endoscopic retrograde cholangiopancreatography; IH: intrahepatic; CBD: common bile duct; FC-SEMS: fully covered self-expandable metal stent. SD Data are expressed as mean +/− standard deviation (min; max), or numbers (percentage).

**Table 4:** Univariate analysis of risk factors associated with recurrence of ABS.

	Odds Ratio	95% Confidence Interval	*p*-Value
Age at LT	0.90	0.78, 1.05	0.2
Gender			
Female	_	_	_
Male	0.04	0.00, 3.10	0.14
LT to diagnosis delay	0.97	0.85, 1.11	0.6
CMV mismatch (R−/D+)			
No			
Yes	1.72	0.13, 23.5	0.7
Type of graft			
Full Graft	13.756.90	0.00, inf	>0.9
Right liver	_	_	_
Left liver	_	_	_
Donor age	1.05	0.97, 1.14	0.2
Pre-LT cold ischemia duration	2.05	0.91, 4.60	0.079
Baseline MRI findings			
Stenosis length	1.01	0.83, 1.23	0.9
Stenosis to hilum short distance	0.73	0.05, 10.7	0.8

LT: liver transplantation; MRI: magnetic resonance imaging.

## Data Availability

Data is unavailable due to privacy or ethical restrictions.
